# Observations in Practice

**Published:** 1876-04

**Authors:** W. L. Lipscomb

**Affiliations:** Columbus, Miss.


### OBSERVATIONS IN PRACTICE.

By Dr. W. L. LIPSCOMB, of Columbus, Miss.

*Gonorrhea a Cause of Sterility in Married Women.* At a session of the Columbus and Lowndes County Medical Society, Gonorrhea was the subject of essay and discussion. I called the attention of the society to the fact that I had noticed in two or three cases, that gonorrhea in married women had been followed by sterility, and asked their opinion as to the connection of the two, as cause and effect. Several members referred to their memory and case books, and we were able to collect five or six well authenticated cases, in which their experience corresponded with mine.

We excluded, promptly, unmarried women from the rule, and suggested the following reasons or hypotheses for the fact:

1st. To produce the sterility, the inner lining of the uterus must be involved in the venereal poison.

2d. The poison prevents the successful attachment of the fœcundated ovum to the uterine walls, or destroys the ovum itself.

3d. The open os, and depressed womb from child-bearing, render the entrance of the poison into the cavity easy and extremely liable.

4th. This condition of the parts does not obtain in virgins or unmarried women, hence they are not liable to intra-uterine gonorrhea.

The field of our experience is too limited for the establishment of a fact. Will the profession assign the suggestion to its proper place, and publish the assignment?

